# Novel role of the dietary flavonoid fisetin in suppressing rRNA biogenesis

**DOI:** 10.1038/s41374-021-00642-1

**Published:** 2021-07-15

**Authors:** Sarah C. Kammerud, Brandon J. Metge, Amr R. Elhamamsy, Shannon E. Weeks, Heba A. Alsheikh, Alexa L. Mattheyses, Lalita A. Shevde, Rajeev S. Samant

**Affiliations:** 1 Department of Pathology, University of Alabama at Birmingham, Birmingham, AL USA; 2 Department of Cell, Developmental, and Integrative Biology, University of Alabama at Birmingham, Birmingham, AL USA; 3 O’Neal Comprehensive Cancer Center, University of Alabama at Birmingham, Birmingham, AL USA; 4 Birmingham VA Medical Center, Birmingham, AL USA

**Keywords:** Breast cancer, Breast cancer, Nucleolus

## Abstract

The nucleolus of a cell is a critical cellular compartment that is responsible for ribosome biogenesis and plays a central role in tumor progression. Fisetin, a nutraceutical, is a naturally occurring flavonol from the flavonoid group of polyphenols that has anti-cancer effects. Fisetin negatively impacts several signaling pathways that support tumor progression. However, effect of fisetin on the nucleolus and its functions were unknown. We observed that fisetin is able to physically enter the nucleolus. In the nucleolus, RNA polymerase I (RNA Pol I) mediates the biogenesis of ribosomal RNA. Thus, we investigated the impacts of fisetin on the nucleolus. We observed that breast tumor cells treated with fisetin show a 20–30% decreased nucleolar abundance per cell and a 30–60% downregulation of RNA Pol I transcription activity, as well as a 50–70% reduction in nascent rRNA synthesis, depending on the cell line. Our studies show that fisetin negatively influences MAPK/ERK pathway to impair RNA Pol I activity and rRNA biogenesis. Functionally, we demonstrate that fisetin acts synergistically (CI = 0.4) with RNA Pol I inhibitor, BMH-21 and shows a noteworthy negative impact (60% decrease) on lung colonization of breast cancer cells. Overall, our findings highlight the potential of ribosomal RNA (rRNA) biogenesis as a target for secondary prevention and possible treatment of metastatic disease.

## Abstract (web summary)

Fisetin, a natural flavanol, can localize to the nucleolus and decrease rRNA biogenesis. It preferentially targets tumorigenic cells and cells with cancer stem-like properties. In combination with the RNA Pol I inhibitor BMH-21, it synergistically inhibits pulmonary metastasis, making this combination an exciting potential therapeutic strategy.

## Introduction

The term “nutraceutical” was first coined by Dr. Stephen De Felice in 1989 [1]. Nutraceuticals have been used to prevent and/or treat diseases and increase wellness since ancient times. Fisetin, a nutraceutical, is a plant-derived polyphenol that is abundant in human food, such as fruits, vegetables, nuts, and wines and has been proven non-toxic [2–4]. Fisetin has garnered significant interest, as it displays a variety of promising biological effects against cancer including antioxidant, anti-inflammatory, and anti-carcinogenic properties [4–7].

**Fig. 1:**
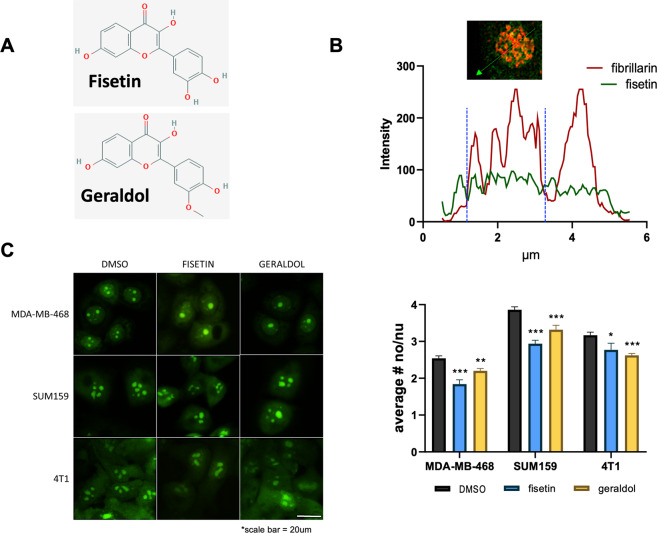
Fisetin localizes to the nucleolus and reduces the number of nucleoli per cell. **A** Chemical structure of fisetin and geraldol acquired from https://pubchem.ncbi.nlm.nih.gov. **B** Fisetin (green) localizes to the nucleolus as evidenced in the representative photomicrograph by its co-localization with fibrillarin (red), a nucleolar marker. The graph indicates the intensity of specific signal (green or red) along a random path (indicated by the green arrow) through the nucleolus. Concurrent intensity increases along 2–3 µm indicates overlapping signal. This observation was noted to be consistent in multiple nucleoli and was repeated in one biologic repeat. **C** MDA-MB-468, SUM159, and 4T1 cells were treated with 5 µM fisetin or 5 µM geraldol for 6 (468 and 159) or 24 h (4T1). Nucleoli were stained using NucleolarID. Images of live cells were captured. Representative photomicrographs are presented. Scale bar = 20 µm. The results are the average of three independent experiments.

Fisetin (3,7,3′,4′-tetrahydroxyflavone) is an orally active biomolecule that has been shown to inhibit or disrupt multiple signaling pathways, including the ERK1/2, mTOR/AKT, MAPK/JNK, NF-κB, and Wnt/β-catenin pathways that tend to signal abnormally in cancer [8, 9]. Treatment of cancer cells with fisetin induces cell cycle arrest, causes cancer cell apoptosis, and inhibits epithelial-to-mesenchymal transition (EMT) [10, 11]. Fisetin modulates the cytoskeletal structure of endothelial cells by increasing microtubule stability and thus displays antiangiogenic activity [12]. It also inhibits the migration of endothelial cells [13]. Touil et al. elucidated pharmacokinetics and in vivo metabolism of fisetin [14]. They discovered that geraldol (3,4′,7-trihydroxy-3′-methoxyflavone) is an active, methoxylated metabolite of fisetin (Fig. 1A). In fact, geraldol was found to be more effective at inhibiting angiogenesis than fisetin [14, 15]. Using label-free two-photon microscopy to visualize it in mouse nerve cells in vitro and in the brains of living mice, Krasieva et al. determined that upon internalization in a cell, fisetin localizes to the nucleolus [16]. Additional studies revealed that other structurally similar flavonols do not localize to the nucleolus, but geraldol, fisetin’s active metabolite does [16].

The nucleolus is a sub-nuclear body that plays critical role in normal cell homeostasis, as well as in the response of the cells to various pathophysiologies [17, 18]. For example, damaged nucleoli play a role in Parkinson’s disease, Alzheimer’s disease, and cardiovascular disease [19–21]. The nucleolus is most widely recognized as the site for ribosome biogenesis but also plays key roles in DNA damage repair, the regulation of gene expression, and response to cellular stress [22–25]. Cancer cells have been found to contain larger and more numerous nucleoli than normal cells [23, 26–29]. Nucleoli form around ribosomal DNA (rDNA) loci that are located in acrocentric chromosomes, (chromosomes 13, 14, 15, 21, and 22). Normal cells under homeostasis, show on average one to two nucleoli. Tumor cells tend to show much higher number. Increased number as well as irregular/enlarged shape of nucleoli in tumor specimen is indicative of poor prognosis [23, 28]. RNA polymerase I (RNA Pol I) carries out a dedicated function of transcribing rDNA to generate ribosomal RNA (rRNA). Effects of flavonoids on RNA synthesis specifically through RNA Pol II has been demonstrated [30]. However, effect of fisetin on impacting RNA Pol I activity remained unexplored. Thus, we sought to investigate the possible effects of fisetin on RNA Pol I activity. Here we present our findings that reveal a negative effect of fisetin on RNA Pol I activity and demonstrate that fisetin acts synergistically with RNA Pol I inhibitor BMH-21 and shows a negative impact on lung colonization of breast cancer cells.

## Materials and methods

### Cell culture

#### Rationale for cell line selection

We recently demonstrated that triple-negative breast cancer (TNBC) cells are more reliant on elevated rRNA biogenesis [31]. Thus, in this study, we use two human TNBCs, SUM159 and MDA-MB-468, and one metastatic murine mammary cancer cell line 4T1. We chose these cells as all these metastasize very readily in vivo. In addition, we have tested MCF7 cells (luminal subtype, ER+ PR+) (Supplementary Fig. 1) to evaluate broader applicability and rigor of our findings.

SUM159 cells were maintained in DMEM/F12 (Thermo Fisher, Waltham, MA) containing 5% FBS (Thermo Fisher), 10 µg/ml insulin (Sigma-Aldrich, St. Louis, MO), and 1 µg/ml hydrocortisone (Sigma-Aldrich). MDA-MB-468 cells were maintained in DMEM/F12 containing 10% FBS. 4T1 cells were maintained in RPMI-160 (Thermo Fisher) containing 5% FBS. MCF10A cells were maintained in DMEM/F12 containing 5% horse serum (Thermo Fisher), 10ug/ml insulin, 1 µg/ml hydrocortisone, 25 ng/ml human epidermal growth factor (hEGF) (Sigma-Aldrich), and 100 ng/ml cholera toxin (Sigma-Aldrich). Normal murine mammary epithelial cells (NMuMG) were maintained in DMEM (Thermo Fisher) containing 10% FBS. MCF7 cells were maintained in DMEM/F12 containing 10% FBS and 10 µg/ml insulin.

### Nucleolar staining

Cells were treated with 5 µM fisetin (Tocris, Bristol, UK), 5 µM geraldol (Extrasynthese, Genay, France), or DMSO (Fisher Scientific, Hampton, NH) as a control. After 6 or 24 h of treatment, cells were stained using NUCLEOLAR-ID^®^ Green detection kit (Enzo Life Sciences, Farmingdale, NY). For each sample, 0.5 µl NUCLEOLAR-ID^®^ Green Detection Reagent and 1 drop NucBlue™ Live ReadyProbes™ Reagent (Invitrogen, Waltham, MA) were diluted in 250 µl 1x phosphate-buffered saline (PBS; Fisher Scientific). This solution was added to each dish with 250 µl regular growth media and placed at 37 °C in humidified incubator at 5%CO_2_ for 10 min before imaging. Images were captured using a Nikon Eclipse Ti-U (Nikon Instruments Inc., Melville, NY). Nucleoli per nucleus were quantified using ImageJ software. Depending on cell type and density, between 15 and 30 fields were counted with an overall average of 30 cells/field. The experiments were repeated (performed twice) with independent passage of respective cell lines.

### Real-time quantitative PCR (RT-qPCR)

Cells were treated as done for nucleolar staining. RNA was harvested after 6 h of treatment using the RNeasy Mini kit (Qiagen, Hilden, Germany) as per the manufacturer’s instructions. cDNA was synthesized from 1 µg of total RNA using the High-Capacity cDNA Reverse Transcription kit (Thermo Fisher) as per the manufacturer’s instructions. cDNA was diluted 1:100, and RT-qPCR was performed using 2X Maxima SYBR Green/ROX Master Mix (Fisher Scientific) and primer sets described previously [32].

Human:

5′ETS 1 for: GAACGGTGGTGTGTCGTT Rev: GCGTCTCGTCTCGTCTCACT

5′ETS 2 for: CAGGTGTTTCCTCGTACCG Rev: GCTACCATAACGGAGGCAGA

Actin for: CATGTACGTTGCTATCCAGGC Rev: CTCCTTAATGTCACGCACGAT

Mouse:

5′ETS 1 for: CACTTTTCTCAGTGGTTCGCG Rev: CAGACGGGAAGGGTATGCAAC

5′ETS 2 for: CTCGAGAGACTCATTGCTTTC Rev: GATGCATGCGACGAGCACAC

Actin for: GGCTGTATTCCCCTCCATCG Rev: CCAGTTGGTAACAATGCCATGT

### Visualization of fisetin and geraldol in the nucleolus

Sixty thousand SUM159 cells were plated on BioCoat Poly-l-Lysine-coated glass coverslips (Corning, Corning, NY) and allowed to adhere for 18 h. Cells were treated with 50 µM fisetin for 30 min at 37 °C. Media was removed, and cells were fixed in 4% paraformaldehyde for 15 min at room temperature. Cells were blocked and permeabilized 1 h at room temperature in 1xPBS containing 5% goat serum (Sigma-Aldrich) and 0.3% triton X-100 (Fisher Scientific) before primary antibody incubation of fibrillarin (1:500, Abcam ab4566, Cambridge, UK) in 1xPBS containing 1% bovine serum albumin (BSA; Fisher Scientific) and 0.3% triton X-100 for 1 h at room temperature. Goat-anti-mouse AlexaFluor 594 (Thermo Fisher; A21125) secondary antibody incubation was as for primary. Coverslips were washed three times in 1xPBS before being mounted on microscope slides using ProLong Diamond Antifade Mountant (Thermo Fisher). Images were captured using a Nikon A1plus. Digital deconvolution and documentation of images were achieved using NIS Elements software (Nikon).

### Nascent rRNA synthesis assay

rRNA synthesis was measured as a readout of 5-Fluorouridine (FUrd) incorporation into nascent rRNA transcripts, as previously described [25, 33]. Briefly, 100,000 cells were seeded onto 22 × 22 glass coverslips (Fisher Scientific) and allowed to attach overnight. The following day, fresh media was added with the addition of 5 µM fisetin, 5 µM geraldol, or DMSO as a control for 6 h at 37 °C in humidified incubator at 5% CO_2_. Following treatment, cells were pulsed with 2 mM FUrd (Sigma-Aldrich) for 15 min at 37 °C in humidified incubator at 5%CO_2_. Following the pulse, cells were immediately fixed with 3.7% formaldehyde diluted in 1xPBS for 10 min at room temperature. Coverslips were subsequently washed and permeabilized with 0.3% triton X-100 in 1xPBS for 15 min at room temperature and blocked in 5% BSA (w/v in 1xPBS). Coverslips were incubated with anti-Brdu (Sigma-Aldrich; B2531) 1:500 and anti-Fibrillarin (Abcam; ab166630) 1:500 diluted in 1xPBS overnight at 4 °C. Coverslips were washed three times in 1xPBS 5 min each at room temperature followed by anti-mouse Alexa Fluor 488 (Thermo Fisher; A11001) or anti-rabbit Alexa Fluor 594 (Thermo Fisher; A11012) secondary antibodies. Following incubation with secondary antibodies, coverslips were washed three times in 1xPBS at room temperature, then mounted using Vectashield Plus with DAPI (Vector Labs, Burlingame, CA). Images were taken with a Nikon Eclipse Ti-U using the same exposure times for all images acquired. Mean Fluorescence Intensity was determined using NIS Elements Advanced Research software analyzing 50 cells across 10 random fields. Representative images are depicted.

### Colony formation assay

Three to five hundred cells were plated per well in 12-well plates. Forty-eight hours after seeding, desired concentration of fisetin and/or BMH-21 (Selleck Chemicals, Houston, TX) was added. Media containing treatment was replaced thrice weekly. After 10–14 days of treatment, the experiments were terminated. Media was removed, and cells with fixed in 4% paraformaldehyde for 15 min at room temperature. Colonies were stained with 0.1% crystal violet in 10% ethanol 5 min and destained in deionized water until water ran clear. Colonies (50 cells or greater) were counted and represented as percent of control. Experiment was done twice in duplicate.

### Nucleolar isolation

Nucleoli were isolated as described previously [34, 35]. Cells were plated and allowed to grow ~48 h to 80% confluence. They were washed, then collected in a minimal volume of 1xPBS, and centrifuged. Cells were subjected to osmotic shock, then lysed with the addition of NP-40 and homogenized using a tight Dounce homogenizer. Nuclei were purified through a 250 mM sucrose cushion, then sonicated. Nucleoli were then purified through a 340 mM sucrose cushion and resuspended in PBS.

### Immunoblotting

Proteins were separated using SDS-PAGE, then transferred to PVDF membranes (BioRad, Hercules, CA). Membranes were blocked with 5% milk in TBS with 0.1% Tween-20, followed by incubation in primary antibody overnight. Primary antibodies used were POLR1A (#24799), p44/42 MAPK (Erk 1/2, #9107), and phospho-p44/42 MAPK (#4370) (all from Cell Signaling, Danvers, MA). After washing in TBST, membranes were incubated in the appropriate secondary antibody. Signal was developed using ECL Prime or ECL Select (Cytiva, Marlborough, MA) and exposed using an Amersham Imager 600 (Cytiva).

### Gene expression analysis

The RNA isolated from SUM159 cells treated with fisetin or geraldol was subjected to Nanostring analysis (Nanostring Laboratory at University of Alabama at Birmingham). A DS-11 Spectrophotometer (DeNovix Inc, Wilmington DE) was used to measure the A_260_/A_280_ ratios to estimate the quality of the RNA. Overall, 100 ng of RNA was used for each reaction. The samples were processed on the NanoString nCounter Flex system using a premade human PanCancer Pathways panel as per the manufacturer’s instructions (NanoString Technologies, Seattle, WA). The panel comprises 770 genes from 13 cancer-associated canonical pathways (MAPK, STAT, PI3K, RAS, Cell Cycle, Apoptosis, Hedgehog, Wnt, Notch, DNA Damage Control, Transcriptional Regulation, Chromatin Modification, and TGF-β), as well as numerous housekeeping genes and positive and negative controls. Gene expression analysis was carried out with nSolver 4.0 software (NanoString Technologies) to directly compare the counts obtained between samples treated with fisetin, geraldol, or vehicle control.

### Functional enrichment analysis

Gene Set Enrichment Analysis (GSEA) was performed using Webgestalt (WEB-based GEne SeT AnaLysis Toolkit) (http://www.webgestalt.org) [36] to determine the normalized enrichment score of enriched pathways. For GSEA, KEGG pathways database was used with minimum number of genes for a category of 15 and number of permutation of 1000. Morpheus tool (https://software.broadinstitute.org/morpheus) was used to generate heatmaps using Log 2 of expression level of all genes and hierarchical clustering for rows and columns using one minus Pearson correlation metric.

### Mammosphere assay

Mammosphere assays were carried out as previously reported [37]. Briefly, 40,000 cells were seeded in each well of an ultra-low attachment plate in the cell line’s base media supplemented with 0.4% BSA (Millipore, Burlington, MA), 25 ng/ml hEGF (Sigma-Aldrich), and 10 ng/ml human basic fibroblast growth factor (Sigma-Aldrich). Plates were monitored daily, media was added as needed, and mammosphere images were captured using the 10x objective on a Nikon Ti-U microscope. Mammosphere area was measured using ImageJ software.

### Evaluation of combination index

4T1 cells (1E5) were plated per well in a 96-well plate. Eighteen hours after plating, cells were treated with varying concentrations of fisetin alone or a combination of fisetin and 0.1 µM BMH-21. The assay was terminated after 48 h of treatment using CyQUANT Direct Cell Proliferation Assay (Thermo Fisher) as per the manufacturer’s instructions. The combination index was calculated using CompuSyn software to determine whether the combination of drugs had synergistic (<1), antagonistic (>1), or no effect (=0).

### Pulmonary metastasis assay

Pulmonary metastasis assays (PuMA) were carried out as previously reported [38]. Two-hundred thousand GFP-expressing 4T1 cells were injected into the tail vein of nude mice. Mice were anesthetized 10 min later and the lungs inflated with a 1:1 solution of 1.2% low-melt agarose (Invitrogen) in culture media 1 [2x M-199 media (Gibco, Waltham, MA) containing 2 µg/ml insulin, 0.2 µg/ml hydrocortisone, 0.2 µg/ml retinyl acetate (Sigma-Aldrich), 1x Penicillin-Streptomycin (Mediatech, Manassas, VA), and 0.225% sodium bicarbonate] using a 27.5 gauge needle inserted into the trachea. After humane euthanasia of the mice, the lungs were removed and placed in 1xPBS containing 1x Penicillin-Streptomycin to allow agarose to solidify. Lungs were sectioned at 1–2 mm and placed on a 2 × 2 × 0.7 cm piece of Surgifoam (Ethicon, Somerville, NJ) that had been soaked in culture media 2 (1x M-199 media containing 1 µg/ml insulin, 0.1 µg/ml hydrocortisone, 0.1 µg/ml retinyl acetate (Sigma-Aldrich), 1x Penicillin-Streptomycin and 0.225% sodium bicarbonate) containing either vehicle control, 1.5 µM fisetin, 0.05 µM BMH-21, or a combination of 1.5 µM fisetin and 0.05 µM BMH-21. Media was replaced thrice weekly, and sections were flipped over with each media change. Pictures were captured using a SMZ800 stereo zoom microscope (Nikon), and mean fluorescent area was determined using NIS Elements software.

## Results

### Fisetin localizes to the nucleolus and reduces the number of nucleoli per cell

Fisetin and its active, methoxylated metabolite, geraldol (Fig. 1A) are fluorophores due to their conjugated ring structure and thus can be imaged using fluorescent microscopy [16]. Fisetin was found to have an emission peak of ~480 nm [16, 39]. These studies were conducted in mouse nerve cells and suggested that fisetin may localize to nucleolus. We used breast cancer cell line SUM159 to evaluate if nucleolar localization is a broader (cell type independent) property of fisetin. Figure 1B depicts nucleolar accumulation of fisetin in SUM159 cells with a concurrent staining of fibrillarin, an exclusively nucleolar marker. As depicted in the graph, green (fisetin) or red (fibrillarin) fluorescence concurrent increase above background at the same location across the plane of the nucleolus of a cell (Fig. 1B). Fibrillarin is localized in the fibrillar center as well as dense fibrillar compartment of the nucleolus. Fisetin seems to show a wider distribution throughout the nucleolus and is not restricted to the dense fibrillar compartment.

We sought to determine the effect of fisetin on nucleoli of cancer cells. We treated human breast cancer cell lines, MDA-MB-468 and SUM159, and mouse mammary tumor line, 4T1, with fisetin. Treated cells exhibited a significant decrease in number of nucleoli per cell compared to control cells (Fig. 1C). A strikingly similar effect was observed in cells treated with the active metabolite of fisetin, geraldol (Fig. 1C).

### Fisetin affects RNA polymerase I activity and rRNA biogenesis

**Fig. 2:**
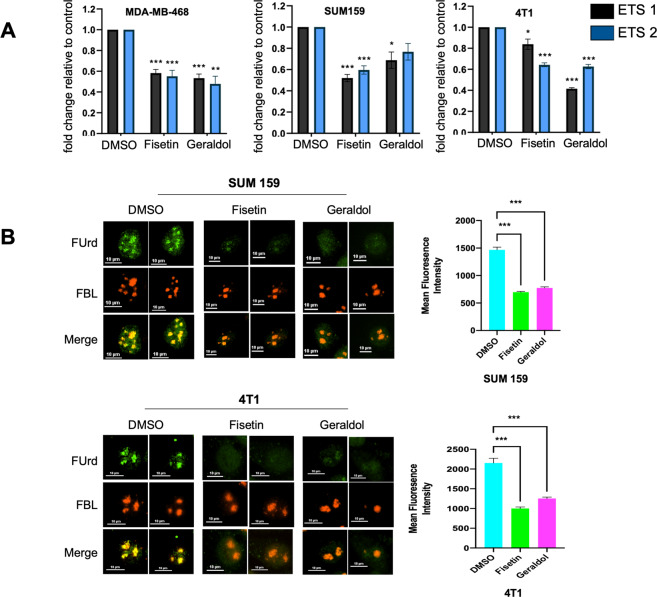
Fisetin affects RNA polymerase I activity and rRNA biogenesis. **A** RNA polymerase I activity is decreased in cells treated with Fisetin or Geraldol. Cells were treated with 5 µM Fisetin or 5 µM Geraldol for 6 h, RNA was harvested, and RT-qPCR was performed. The results are the average of two independent experiments performed in triplicate. Errors bars represent standard error of the mean (***p < 0.001, **p < 0.01, and *p < 0.05). **B** Decreased FUrd incorporation in SUM159 or 4T1 treated with 5 µM Fisetin or 5 µM Geraldol 6 h. Cells were treated then pulsed with 2 mM 5 Flourouridine(FUrd). FUrd incorporation (green) in combination with Fibrillarin (red) was used to determine rRNA biogenesis. Graphs indicate fold change in mean fluorescence intensity, 50 cells quantified in each group. ***p < 0.0001.

Nucleoli form around actively transcribing rDNA. Thus, reduction in the number of nucleoli per cell may indicate a reduced activity of RNA Pol I. Therefore, we analyzed RNA Pol I activity in cells treated with both fisetin and geraldol. 47S rRNA is the precursor that gets spliced into 5.8S, 18S, and 28S rRNA. In this process, the externally transcribed spacer regions (ETS) are spliced out from the precursor rRNA and are very rapidly degraded. Thus, their levels are reflective of RNA Pol I activity [32, 40]. We observed that RNA Pol I activity, as measured by 5′ETS levels, was significantly decreased across all three cell lines (Fig. 2A). In addition, we evaluated hormone receptor positive breast cancer cells, MCF7 (Supplementary Fig. 1). With both fisetin as well as geraldol, these cells also showed an effective reduction in the number of nucleoli per cell and reduced activity of RNA Pol I.

To obtain a more comprehensive estimate of this activity using an independent technique, we assessed the effect of both fisetin and geraldol on nascent RNA synthesis. For a very short duration, predominant nascent RNA species synthesized in a cell is rRNA. Cells were pulsed with FUrd for 15 min, and the incorporation of FUrd was visualized and estimated. Majority nascent RNA synthesis is seen in the nucleolus. We noticed about 30–45% drop in FUrd incorporation in SUM159 as well as 4T1 cells upon treatment with fisetin and geraldol. This clearly indicated that fisetin and geraldol reduced rRNA biogenesis (Fig. 2B). Overall, our observations thus far indicate a clear negative effect of fisetin and geraldol on rRNA biogenesis.

### Cancer cells show greater sensitivity to growth inhibition by fisetin

Fisetin has been shown to target tumorigenic cells more readily than non-tumorigenic cells in multiple cancers. For example, the growth of non-small cell lung cancer cells was inhibited while that of normal bronchial epithelial cells was not [41]. Fisetin induced apoptosis and inhibited the growth of prostate cancer cells but had little effect on normal prostate epithelial cells [42]. Pancreatic, colon, breast, and prostate cancer cells were shown to have significantly lower GI_50_ (50% of maximal growth inhibition of cell proliferation) values for fisetin than normal cells [43].

**Fig. 3:**
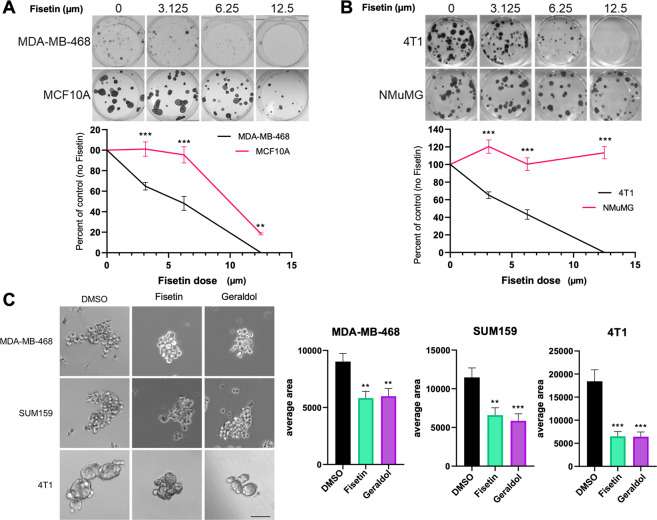
Cancer cells show greater sensitivity to growth inhibition by fisetin. **A** Human (MDA-MB-468) and **B** Murine (4T1) breast cancer cells treated with fisetin show a decrease in the formation or colonies relative to normal human (MCF10A) and mouse (NMuMG) cells. Representative photomicrographs are shown. The corresponding graphs represent colonies (percent of control) at a respective concentration of fisetin. The results are the average of two independent experiments performed in triplicate. **C** Fisetin and geraldol inhibit the formation of mammospheres in MDA-MB-468, SUM159, and 4T1 cells. The results are the average of two independent experiments performed in triplicate. Scale bar = 100 µm. Error bars represent standard error of the mean (***p < 0.001 and **p < 0.01).

Given our findings that RNA Pol I activity is significantly reduced by fisetin, we evaluated fisetin’s impact on the growth of cancer cells. We performed a colony formation assay with human and mouse non-tumorigenic and tumorigenic cell lines. The tumorigenic cell lines showed a dose-dependent inhibition of colony formation (human MDA-MB-468 and mouse 4T1), while non-tumorigenic human breast cells (MCF10A) and NMuMG were minimally affected (Fig. 3A, B).

Cancer stem cells pose a significant challenge as they are responsible for drug-resistant recurrence and metastatic dissemination. Targeting stem cells can diminish self-renewal capability and inhibit tumor progression and relapse. Mammosphere-formation assay is a broad indicator of stem cell activity in breast cancer cell lines. We found that both, fisetin and geraldol effectively inhibited the formation of mammospheres from MDA-MB-468, SUM159, and 4T1 cells (Fig. 3C).

### Fisetin and Geraldol downregulate MAPK signaling

**Fig. 4:**
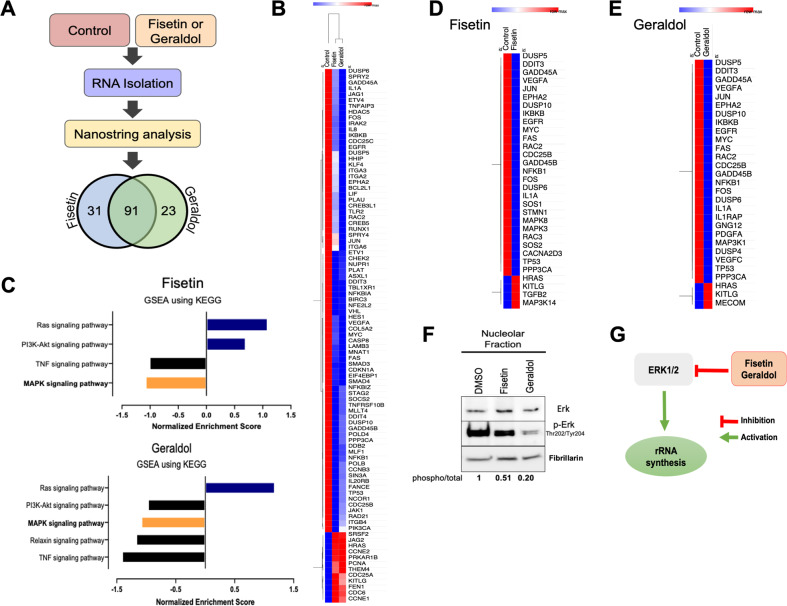
Fisetin and geraldol downregulate MAPK signaling. **A** Schematic of experimental approach and results of Nanostring analysis. Venn diagram represents significantly changed expression of transcripts in fisetin and geraldol groups, respectively. **B** Clustering of overlapping 91 differentially changing transcripts for fisetin and geraldol compared to the control. **C** Signaling pathways enriched in response to Fisetin or Geraldol analyzed by gene set enrichment analysis (GSEA) using Kyoto Encyclopedia of Genes and Genomes (KEGG). **D** The list of all genes annotated to MAPK signaling in KEGG database was used to identify the genes involved in MAPK signaling that changed their expression in response to Fisetin and **E** Geraldol. **F** Western blot analysis of levels of total ERK and P- ERK from nucleolar lysates of SUM159 cells treated independently with Fisetin or Geraldol. The numbers below the lanes are the densitometric estimation of signal intensity relative to the control (DMSO). **G** Schematic representing negative impact of fisetin and geraldol on Erk pathway leading to reduced rRNA synthesis.

We used Nanostring nCounter^®^ PanCancer Pathways Panel to identify the changes in gene expression following treatment with fisetin and geraldol. For this, we treated highly metastatic TNBC cells SUM159 cells, independently with both fisetin and geraldol. Expression of 145 genes altered upon treatment with fisetin or geraldol, with an overlap of 91 genes between the two drugs (Fig. 4A). The overlapping genes clustered distinctly for fisetin and geraldol, displaying a very similar pattern of gene expression (Fig. 4B). As a confirmation, we checked protein levels of two genes relevant to this study POLR1A (the largest subunit of the RNA Pol I complex) and ERK 1/2. Both POLR1A and ERK 1/2 showed a noticeable decrease upon treatment with fisetin or geraldol (Supplementary Fig. 2).

These data were analyzed by GSEA using Kyoto Encyclopedia of Genes and Genomes (KEGG) Pathway database to identify signaling pathways enriched in response to fisetin or geraldol. As shown in Fig. 4C, MAPK signaling, among other pathways, was significantly negatively enriched in fisetin and geraldol treated cells.

To identify the players in MAPK signaling that are changing in response to fisetin and geraldol, list of all genes annotated to MAPK signaling in KEGG database was used. Fisetin downregulated the expression of 27 genes (Fig. 4D) involved in MAPK signaling (KEGG PATHWAY: hsa04010) including MAPK3 (ERK1), MAPK8 (JNK1), and FOS (AP-1), whereas it upregulated HRAS and TGFB2 expression. Geraldol showed a similar pattern of downregulating the expression of 26 genes (Fig. 4E) involved in MAPK signaling including MAP3K1 (MEKK1) and FOS (AP-1).

Interestingly, ERK regulates ribosome biogenesis and has multiple intracellular locations including the nucleolus [25, 44]. We analyzed nucleolar extracts from SUM159 cells (control as well as treated with fisetin and geraldol) for levels of active nucleolar ERK. As seen in Fig. 4F, we observed that both, fisetin and geraldol starkly reduced p-ERK levels. Geraldol was much more effective than fisetin in this activity. Based on our observations, we contend that fisetin negatively impacts nucleolar ERK activity and reduces ribosome biogenesis (Fig. 4G).

### Fisetin inhibits functional attributes of malignant mammary tumor cells

**Fig. 5:**
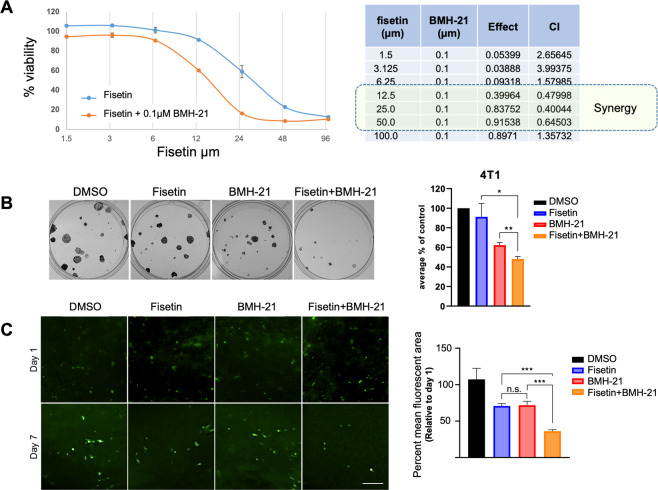
Fisetin inhibits functional attributes of malignant mammary tumor cells. **A** Determination of CI (combination index) of Fisetin and BHM-21. Percent viability of 4T1 cells is determined using various concentration of Fisetin (alone, blue curve) or in combination with 0.1 µM BHM-21(orange curve). The adjacent table shows the determination of CI for each combination. **B** 4T1 cells treated with a combination of fisetin and BMH-21, an RNA pol I inhibitor, show a significant decrease in colony formation relative to control and single-drug treatment. Data are representative of two independent experiments performed in duplicate. **C** Co-treatment of 4T1 cells in ex vivo PuMA assay with fisetin and BMH-21 inhibits the formation of pulmonary metastases significantly more than single-drug treatment or control. Scale bar = 250 µm. Errors bars represent standard error of the mean (***p < 0.001, **p < 0.01, and *p < 0.05).

Our observations thus far revealed a negative impact of fisetin on RNA Pol I. We hypothesized that co-treating cancer cells with fisetin and an RNA Pol I inhibitor would elicit a synergistic effect that would allow for effective tumor cell killing at lower doses of each drug. BMH-21 inhibits RNA Pol I and selectively kills tumor cells relative to normal cells [32, 45]. We determined CI (combination index) of fisetin and BHM-21. CI value (<1) is considered synergistic. Fisetin showed synergy with BHM-21 at multiple concentrations with CI of 0.4–0.6 (Fig. 5A).

We analyzed the colony formation ability of 4T1 cells treated with very low doses of fisetin (1.5 µM) or BMH-21(0.05 µM) and a combination of both. We observed that BMH-21 was more effective than fisetin. However, the combination was strikingly effective in reducing in colony formation ability compared to the single-dose treatment groups (Fig. 5B).

Lung is one of the preferred sites of breast cancer metastasis. PuMA is a powerful ex vivo assay that allows the evaluation of metastatic out growth in lungs. PuMA is also a very effective tool to evaluate the functional relevance of drug combination [38, 46, 47]. We found that combined treatment with fisetin and BMH-21 significantly inhibited pulmonary metastasis compared to control and single-drug treatment (Fig. 5C). Cumulatively our observations suggest that fisetins and BMH-21 combination will effectively and preferentially target tumor progression and metastatic growth of breast cancer cells by targeting RNA Pol I activity.

## Discussion

Nutraceuticals have been a focus of rigorous studies in cancer biology due to the underlying promise of moderate-to-no side effects. Some of the leading nutraceuticals that show adverse effects on cancer cells include curcumin, quercetin, resveratrol, and polyphenols like epigallocatechin gallate [48, 49]. Curcumin has been shown to impair cell viability, induce apoptosis, inhibit migration and invasion, and deactivate the MAPK pathway [50–52]. Quercetin also induces apoptosis and inhibits migration and invasion, in addition to blocking EMT through inactivation of ß-catenin [53, 54]. As with curcumin and quercetin, resveratrol induces apoptosis, along with inhibiting migration and invasion [55, 56]. It also inhibits the NF-κB and MAPK pathways [57, 58]. Similar to these nutraceuticals, fisetin has broad signaling impacts on target cells [10, 41, 42, 59, 60].

The nucleolus is an intra-nuclear, non-membranous compartment formed as multiphase liquid phase condensate that is centrally involved in maintaining cellular homeostasis under normal and disease conditions [17, 18]. The nucleolus forms around nucleolar organizer regions [61]. It is the sole site of ribosome biogenesis and thus is at the core of the ability of a cell to dynamically respond to its surrounding through changes in cellular protein translation, and thus overall protein composition [62–64]. RNA Pol I transcribes the rDNA to produce rRNAs. rRNAs are then processed and assembled with ribosomal proteins. Thus, the nucleolus is a vital component for the cell to be able to meet the dynamic demands of critical activities that also are characteristics of cancer cells [63, 65]. Depending on cell type and state, the nucleolus may harbor up to a few thousand proteins. A large portion of these proteins are involved in ribosome biogenesis and are key to regulating the cellular stress response [62, 66–68]. While ribosome biogenesis has long been known to be a primary function of the nucleolus, more recent studies have shown the nucleolus plays a role in numerous other cellular processes, including cell cycle regulation, stress response, senescence, and the formation of ribonucleoprotein particles [23]. Nucleolar number is indicative of metabolic activity of the cells. Highly metabolically active cells, such as proliferating cells, tend to show increased numbers of nucleoli. Sustained elevated ribosome biogenesis is one of the classical hallmarks of aberrant cell growth. With its distinct pathological features in malignant cells, the nucleolus has attracted recent attention as a novel drug target [31, 69, 70]. Many structurally diverse molecules have been reported to directly or indirectly perturb the dynamic functionality of the nucleolus [69]. Thus, targeting the nucleolus by targeting RNA Pol I and rRNA biogenesis is at the cutting edge of drug discovery [28, 70]. However, very few drugs have been shown to physically home to the nucleolus and impact its functionality.

Inhibition of RNA Pol I has been an up-and-coming, promising therapeutic approach for treating cancer [40]. One of the well-studied RNA Pol I inhibitors, CX-5461, acts through multiple mechanisms, including the activation of the DNA damage response, and is currently in a phase I clinical trial [71]. Another leading RNA Pol I inhibitor, BMH-21, was first identified as an activator of p53 and later found to be effective in TP53 null and mutant cell lines, indicating that it works upstream of p53 [32, 45]. It was found to intercalate with DNA and inhibit RNA Pol I by inducing proteasome-mediated degradation of RPA194, the largest subunit on the RNA Pol I complex [32]. Thus, it was demonstrated as a selective killer of tumor cells compared to normal cells [45].

Our work presented herein highlights the unique ability of fisetin as a nutraceutical that is able to physically localize to the nucleolus. We also demonstrate that fisetin impairs rRNA biogenesis by reducing RNA Pol I activity. Overall, our investigations revealed negative impacts of fisetin and geraldol on rRNA biogenesis. The details of the impacts of inhibitory effect of flavonoids on DNA-dependent DNA and RNA polymerases have been noticed before [25, 30]. These previous studies were more focused on RNA Pol II. However, recent landmark studies by Abraham et al. reveal that RNA polymerases II can also intervene in ribosome biogenesis [72]. Thus, many complex intricacies of the activity(s) of these flavonoids still remain to be described.

Fisetin has inhibitory effects on cyclin-dependent kinase 6 (CDK6). Lu et al. have solved the structure of a complex between and fisetin and CDK6 [73]. This suggests that fisetin may inhibit growth through inhibition of cell division. We noticed the inhibitory effects of fisetin and geraldol on colony formation. However, one-to-one correlation of this activity of these flavonoids with a specific molecular mechanism is still elusive. Proteomic profiles of cells grown in 3D low attachment culture vs. monolayer showed an increase in proteins related to ribosome biogenesis in the cells grown in 3D culture, suggesting that maintenance of stemness warrants increased ribosome biogenesis [74]. Breast cancer stem cells arguably are critical to its metastasis [75]. In this context, the significant impact of fisetin on mammosphere formation that we have described is notable. It is important to note that in our studies geraldol appears to be just as effective as fisetin in impacting rRNA biogenesis. Other reports from the literature indicate that geraldol exhibits low cytotoxicity, with no effect on primary cell types [14, 76]. Geraldol has been advocated to also be of potential therapeutic value for NMO (Neuromyelitis optica) drug development and may usher a new class of antitumor drugs [14, 77]. Thus geraldol may be a promising candidate for further drug development.

ERK signaling controls growth factor-regulated transcription activation of ribosome biogenesis [35, 78–80]. Stefanovsky et al. demonstrated that ERK phosphorylates upstream binding factor (UBF), a transcription activator of RNA Pol I and that this phosphorylation is important for the activity of UBF [78]. We observe that fisetin inhibits ERK activation. Thus, inhibition of nucleolar ERK signaling may be an avenue to target the dependence of cancer cells on ribosome biogenesis.

Multiple-targeted agents are under evaluation in clinical trials, including EGFR and HER2 inhibitors and agents that alter crucial signaling circuits, such as RAS/MEK/ERK, phosphatidylinositol-3-kinase/Akt/ mammalian target of rapamycin, etc [81]. However, all of these strategies have significant side effects and thus warrant stringent exclusion criteria or relevant stratification of patients. Moreover, targeting occult, dormant metastases that disseminate fairly early in the course of disease progression pose a persistent threat to the patient. A combination of fisetin, which impairs stemness and impacts RNA Pol I activity, with BMH-21, permits effective breast cancer cell killing at significantly reduced dose of each drug. And this, we submit, is a very promising option for potential therapeutic strategy. Since both these compounds have minimal effects on normal cells, the combination may not just be a treatment strategy but may also be a metastasis prevention approach.

## Supplementary information

Supplementary material
