# Supplementary material for: Novel role of the dietary flavonoid fisetin in suppressing rRNA biogenesis
Source: Lab Invest. 2021 Jul 15;101(11):1439–48. doi: 10.1038/s41374-021-00642-1 (PMC8510891; doi:10.1038/s41374-021-00642-1)
Supplement: Supplementary file 1 — Supplementary material [file 41374_2021_642_MOESM1_ESM.pdf]

Supplementary Figure Legends:

Supplementary Figure 1: Fisetin and Geraldol reduce the number of nucleoli per cell and RNA Pol I activity in MCF7

A. In hormone receptor positive MCF7 human breast cancer cells were treated with 5uM fisetin or 5uM geraldol for 24 hours. Nucleoli were stained using NucleolarID. Images of live cells were captured. Representative photomicrographs are presented. Scale bar = 20µm. The results are the average of three replicates.

B. MCF7 cells were treated with 5µM fisetin or 5µM geraldol for 24 hours, RNA was harvested, and RT-qPCR was performed. The results are from three replicates.

Supplementary Figure 2: Analysis of protein expression from SUM159 cells treated with Fisetin or Geraldol.

Total cell lysate from SUM159 treated with 5µM fisetin or 5µM Geraldol for 24 hr was analyzed by Western blot for levels of Erk1/2 and POLR1A

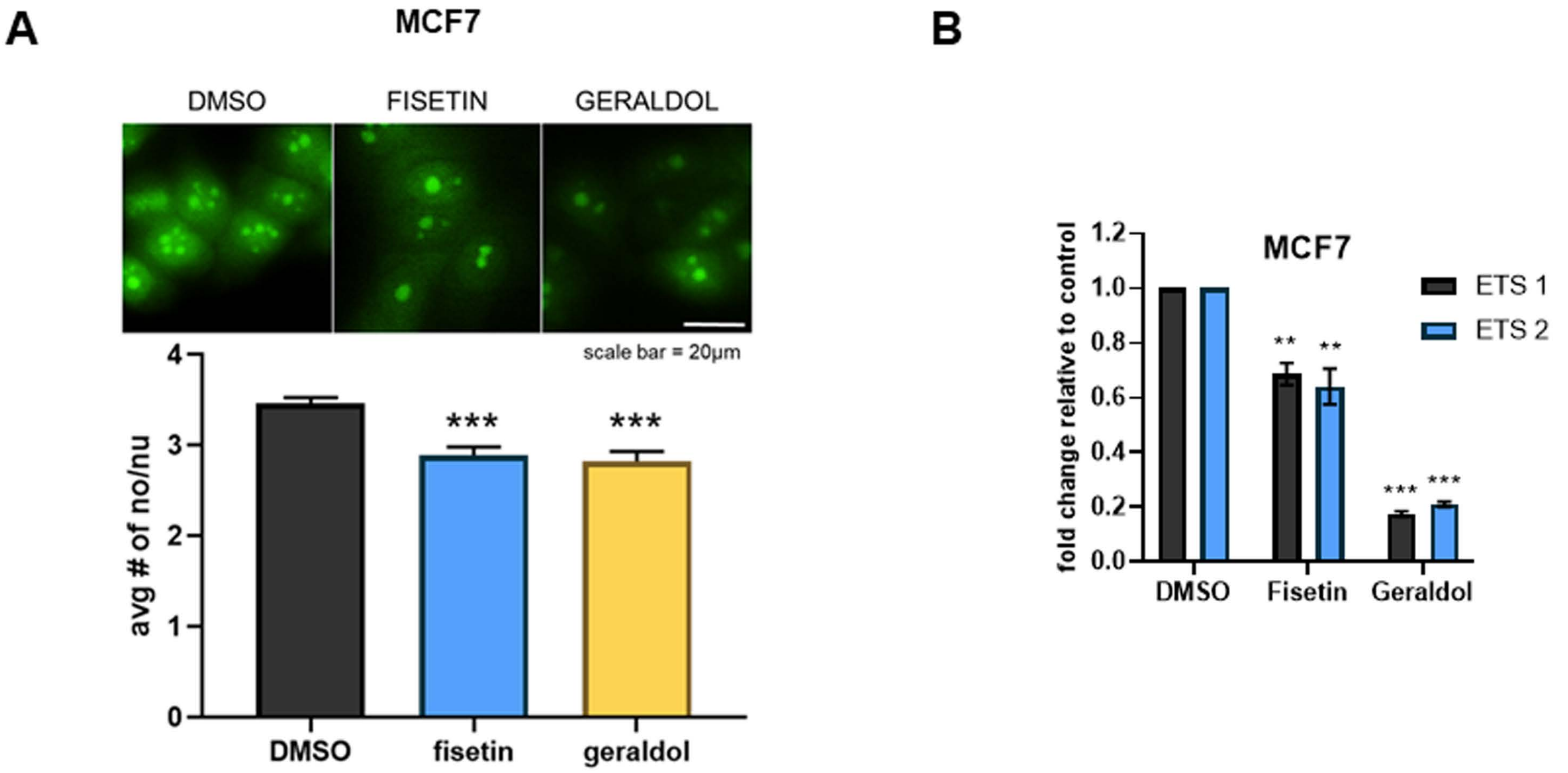

PolI activity RTQ 5-ETS in T47D and MCF7 6 hours post treatment 5uM Fisetin or 5uM Geraldol

Supplementary Figure 1

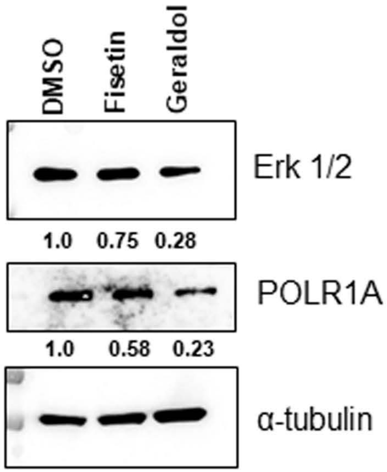

Supplementary Figure 2
